# Text-messaging to promote smoking cessation among individuals with opioid use disorder: quantitative and qualitative evaluation

**DOI:** 10.1186/s12889-022-13008-z

**Published:** 2022-04-06

**Authors:** Divya Shankar, Belinda Borrelli, Vinson Cobb, Lisa M. Quintiliani, Tibor Palfai, Zoe Weinstein, Katia Bulekova, Hasmeena Kathuria

**Affiliations:** 1 Pulmonary Center, Department of Medicine, Boston University Medical Center, 72 East Concord Street, R304, Boston, MA USA; 2 Boston University, Henry M. Goldman School of Dental Medicine, Boston, MA USA; 3 Section of General Internal Medicine, Boston Medical Center and Boston University School of Medicine, Boston, MA USA; 4 Psychological and Brain Science, Boston University, Boston, MA USA; 5 Clinical Addiction Research and Education (CARE) Unit, Section of General Internal Medicine, Boston Medical Center and Boston University School of Medicine, Boston, MA USA; 6 Research Computing Services (RCS) group, Information Services & Technology, Boston University, Boston, MA USA

**Keywords:** Tobacco dependence treatment, Tobacco use disorder/ therapy, Opioid use disorder, Mobile health interventions, Text messaging, Substance-related disorders, Social determinants of health

## Abstract

**Introduction:**

Individuals with opioid use disorder (OUD) who smoke cigarettes have high tobacco-related comorbidities, lack of access to tobacco treatment, lack of inclusion in smoking cessation trials, and remain understudied in the mobile health field. The purpose of this study was to understand patients’ with OUD perceptions of 1) text message programs to promote smoking cessation, 2) content and features to include in such a program, and 3) how message content should be framed.

**Methods:**

From December 2018 to February 2019, we recruited 20 hospitalized individuals with a concurrent diagnosis of OUD and tobacco dependence at Boston Medical Center (BMC), the largest safety-net hospital in New England. We surveyed participants’ cell phone use, their interest in a text message program to promote smoking cessation, and their reactions to and ratings of a series of 26 prototype texts. We then conducted open-ended interviews to elicit content and suggestions on how text message interventions can improve motivation to increase smoking cessation among individuals with OUD. The interviews also included open-ended inquiries exploring message ratings and message content, inquiries about preferences for message duration, frequency, and personalization.

**Results:**

Quantitative analysis of questionnaire data indicated that the majority of participants owned a cell phone (95%, 19/20). Most participants (60%, 12/20) reported that they would be interested or very interested in receiving text messages about smoking cessation. Text messages about the health benefits of quitting were rated the highest among various categories of text messages. Qualitative analysis showed that almost every participant felt that text messages would help motivate smoking cessation given the support it would provide.

**Conclusions:**

This study demonstrates that individuals with OUD who smoke cigarettes perceive that a text message program designed to promote smoking cessation would motivate and support smoking cessation efforts. Our findings demonstrate that such a program is feasible as participants own cell phones, frequently send and receive text messages, and have unlimited text message plans. Findings from this study provide valuable insight into content and features to include when developing text message programs to address barriers to smoking cessation in individuals who have OUD and smoke cigarettes.

## Background

Despite declining rates of smoking nationwide, tobacco use rates among patients with opioid use disorder (OUD) continue to rise [1]. Patients with OUD are two to four times more likely to smoke cigarettes than the general population. The prevalence of smoking cigarettes among individuals with OUD has been found to be as high as 95% in patients not on opioid agonist therapy (OAT) and 84% in patients treated with OAT (e.g., methadone) [2, 3]. Not only do those with OUD smoke more heavily, they also have a higher level of nicotine dependence than those without OUD [4].

While some may be hesitant to recommend tobacco cessation in patients with OUD because it is perceived as too difficult [5], tobacco cessation could have a beneficial effect on cessation of opioid use, given the overlapping physiological pathways involved in both nicotine and opioid use [6–8]. Surveys of individuals in treatment programs have demonstrated that many are interested in quitting their tobacco use [5]. However, little is known about the types of intervention approaches that would match this population’s needs and preferences, particularly given the many barriers to connecting these individuals with treatment, including limited social support, unmet social determinant of health (SDOH) needs, and negative patient experiences with the healthcare system [9, 10].

The efficacy of text message interventions for smoking abstinence is well established. Given the near-universal use of cell phones across all ethnic, income, and educational levels in the United States, text message interventions eliminate many traditional barriers to accessing treatment [11, 12]. Individuals with OUD who smoke meet the definition of an underserved population [13] because of their high smoking rates, tobacco-related comorbidities, lack of access to treatment, and lack of inclusion in prospective smoking cessation trials. Prior work has demonstrated that individuals with OUD are open to incorporating personalized digital health interventions to assist them with their OUD [14–16]. Given the success of text message interventions in other underserved smoking populations and the acceptability of digital health interventions in those with OUD, we sought to understand whether such an intervention targeting smoking cessation could be successful in individuals with OUD.

The purpose of this study was to 1) understand patient perceptions of text message programs to promote smoking cessation, 2) determine content and features to include in such a program, and 3) understand patient preferences in how message content should be framed.

## Methods

### Enrollment

From December 2018 to February 2019, we recruited a convenience sample of 20 hospitalized individuals with a concurrent diagnosis of OUD and tobacco dependence at Boston Medical Center (BMC), the largest safety-net hospital in New England. Potential participants were identified from a list of hospitalized patients who triggered an automatic consultation to the Tobacco Treatment Consult Service based on “current smoking” status in the electronic health record (EHR), which is the standard of care at BMC [17]. We then reviewed their medical charts for a documented ICD-10 diagnosis of OUD. Patients were eligible if they met the initial screening criteria: current users of cigarettes and a diagnosis of OUD (including those with OUD on OAT). Participants were excluded if they were under the age of 18, cognitively impaired, or non-English speaking. Seventy-two patients met initial chart screening criteria; thirty-five were not approached because they were not available for interview by study staff. Participants were provided with information on the interview study as well as the goals of text message program development. Twenty (54%, 20/37) agreed and gave informed consent to participate in the study; participants received a $25 gift card for participation.

### Measures

#### Questionnaires

Questionnaires were administered verbally by study staff to participants. Assessments included demographics (Table 1), substance use and frequency of use (unprescribed opioids, alcohol, cocaine, marijuana, unprescribed benzodiazepines, methamphetamines, hallucinogens, ecstasy/MDMA), tobacco), treatment with methadone or suboxone, and comorbid mental health disorders. Cigarette use characteristics (e.g. frequency of use, prior quit attempts; Table 2) were collected and the Fagerstrom Test for Nicotine Dependence [18] was administered. We surveyed participants on prior smoking cessation methods utilized, whether these were effective, if participants would be willing to try them again, and their timeframe to quit smoking. Participants were also asked about the use of e-cigarettes and other tobacco products. Importance and motivation to quit were assessed on a five-point scale (very important/motivated, important/motivated, neutral, slightly important/motivated, not at all important/motivated). Participants’ cell phone use characteristics were surveyed, and their interest in a text message program and/or interactive text message program (which would allow them to answer questions and receive programmed messages based on their responses), was assessed on a four point-scale (very interested, interested, a little interested, not at all interested).

We then surveyed participant’s ratings of a series of prototype text messages. Participants were asked to read 26 messages (Table 3). Messages were from 3 sources: (1) the National Cancer Institute’s Smokefree TXT [19], (2) content adapted from prior work by Borrelli et al. [20], (3) novel messages developed by the study team. The text messages have a Flesch-Kincaid grade equivalent result of 5.6, indicating that the text messages are at the reading level of an average student in fifth grade [21]. Novel messages centered around the theme of how tobacco cessation may impact recovery from other substances, specifically how patients who quit smoking have higher success in quitting other substances [5]. We also created messages that provided information on the safety and efficacy of medications as well as behavioral tips on how to use medication. Participants rated each message (yes/no) as to whether it was (1) helpful, (2) likeable, and (3) motivating to quit. Additional survey questions adapted from the Barriers to Quitting Smoking in Substance Abuse Treatment (BQS-SAT) scale [22], a measure of the perceived importance of barriers to quitting smoking in substance use treatment, were added to the interview protocol halfway through the study to further probe challenges to stopping smoking while quitting other substances.

#### Qualitative interviews

After questionnaires were administered, study authors (research team members and no clinical relationships with participants) conducted semi-structured, exploratory interviews with the 20 participants. Responses were audio-recorded with participants’ permission. We used the Social Contextual Model to develop our interview guide. This model provides a useful framework for categorizing the social context (e.g. SDOH) that influences health behaviors [23, 24]. Our interview guide was designed to identify modifiable aspects of social context and elicit participant suggestions on how text message interventions can best address these factors to improve motivation to increase smoking cessation among individuals with OUD. The interviews also included open-ended inquiries exploring message ratings and content, preferences for message frequency, duration, and personalization, experiences with cessation medications, and preferences among sample message types (e.g., preference for information on health risks and benefits or motivational messages).

#### Data analyses

Responses from the questionnaire were collected and managed using REDCap (Research Electronic Data Capture) tools hosted at Boston University, CTSI 1UL1TR001430. REDCap is a secure, web-based application designed to support data capture for research studies, providing 1) an intuitive interface for validated data entry; 2) audit trails for tracking data manipulation and export procedures; 3) automated export procedures for seamless data downloads to common statistical packages; and 4) procedures for importing data from external sources [25, 26].

Basic descriptive statistics were calculated using the Statistical Program for Social Sciences v18 to summarize responses to the questionnaire. For the qualitative analyses, interviews were transcribed verbatim from audio recordings. We analyzed transcripts using both deductive and inductive content analysis [27, 28]. For deductive analysis, data were mapped to constructs from the Social Contextual Model to identify barriers and modifiable factors. For inductive analysis, we performed unstructured coding of transcripts to allow for identification of new themes. We developed a preliminary codebook and independently reviewed all transcripts and revised and added codes until the team reached consensus on codes and summary categories. We finalized conceptual categories, grouped themes in each category, and identified specific quotes that best highlighted individual themes. Supporting statements are identified by participant number. The Boston University Medical Campus Institutional Review Board approved the project.

## Results

### Questionnaires

#### I. Demographics

Participant demographics are reported in Table 1. Most were Medicaid-insured (90%, 18/20), unemployed (90%, 18/20), and currently homeless (60%, 12/20). Nine (45%, 9/20) reported active opioid use. Thirteen (65%, 13/20) were receiving medication-assisted treatment with methadone or buprenorphine; 2 individuals (15%, 2/13) reported concomitant unprescribed opioid use. Use of other substances was common with 13 (65%, 13/20) using cocaine, 7 (35%, 7/20) using alcohol heavily (5 or more drinks for men daily; 4 or more drinks for women daily), and 9 (45%, 9/20) using marijuana. Seventeen participants (85%, 17/20) self-reported depression and/or anxiety, and 6 participants (30%, 6/20) self-reported bipolar disorder.

**Table 1:** Demographic characteristics of participants

Demographic Characteristic	Participants (*n* = 20)
**Age, mean (range)**	42.2 (26-60)
**Male**	10 (50%)
**Race**	
White	11 (55%)
Black/African American	6 (30%)
Mixed	2 (10%)
Other	1 (5%)
**Hispanic/Latino Ethnicity**	6 (30%)
**Insurance Status**	
Medicaid (MassHealth)	18 (90%)
Private insurance	2 (10%)
**Education Level**	
Grades 9-11	7 (35%)
Graduated high school	2 (10%)
GED	5 (25%)
Some College	4 (20%)
Technical School	1 (5%)
Associate degree from college	1 (5%)
**Homelessness**	12 (60%)
**Marital Status**	
Married	1 (5%)
Divorced/separated	4 (20%)
Living together	1 (5%)
Never married	14 (70%)
**Unemployed**	18 (90%)
**Yearly household income before taxes**	
$0-$14,999	16 (80%)
$15,000-$34,999	2 (10%)
$35,000-$74,999	2 (10%)

#### II. Tobacco product use and history

Tobacco use characteristics and quit beliefs for participants are reported in Table 2. Three participants (15%, 3/10) were ready to quit within 1-6 months and 7 participants (35%, 7/20) were ready to quit within 1-5 years. Ten participants (50%, 10/20) were not ready to set a quit date. Of the 18 participants who had tried the nicotine patch before, 10 (56%, 10/18) said it was helpful. Of the 10 participants who had tried pharmacotherapy (Chantix and/or Wellbutrin), 4 (40%, 4/10) said it was helpful. Only two (10%, 2/10) had tried text message programs to quit previously, and they both said this had been helpful in the past and that they would want to try this method again.

**Table 2:** Tobacco use characteristics and beliefs of participants

	Participants (*n* = 20)
Fagerstrom score (tobacco dependency rating) high or very high ^a^	9 (45%)
Daily cigarette use	17 (85%)
Believed that it is “important” or “very important” to quit smoking ^b^	12 (60%)
“Motivated” or “very motivated” to quit smoking ^b^	7 (35%)
Attempted to quit in past year	9 (45%)
Prior use of nicotine patch to quit smoking	19 (90%)
Prior use of medication cessation	10 (50%)
*Varenicline*	4 (20%)
*Bupropion*	6 (30%)
Prior use of text message programs to quit	2 (10%)

^a^ Scores for the Fagerstrom Test for Nicotine Dependence [18] range from 0 to 10, with higher scores indicating a more intense physical dependence on nicotine; high or very high dependence corresponds to a score of 8-10

^b^ Importance and motivation to quit were assessed on a five-point scale (very important/motivated, important/motivated, neutral, slightly important/motivated, not at all important/motivated)

Eleven participants (55%, 11/20) reported dual tobacco product use in the past month: 8 participants (40%, 8/20) reported concurrent use of e-cigarettes; 1 (10%, 1/20) participant reported concurrent use of chewing tobacco or snuff; and 2 (10%, 2/20) participants reported concurrent use of other, unspecified tobacco products in the past month.

#### III. Feasibility and acceptability of a text message program

Questionnaire data indicated that the vast majority of participants reported owning a cell phone (95%, 19/20), of which 18 (95%, 18/19) used ‘smart phones.’ All 19 cell phone owners had unlimited text message plans and could connect to the internet on their handheld devices. Twelve participants (60%, 12/20) reported sending and receiving more than 10 texts per day. Eighteen participants (90%, 18/20) had previously downloaded a mobile application onto their cell phones.

The majority of participants reported that they would be interested or very interested in receiving text messages about smoking cessation (60%, 12/20). Seven participants reported that they would be a little interested and 1 participant reported that she was not at all interested in receiving text messages about smoking cessation. Fifteen participants (75%, 15/20) were interested or very interested in an interactive text message program designed to help them quit smoking or become more motivated to quit smoking.

#### IV. Text message content

Participants were read twenty-six sample text messages and ratings and reactions to these were recorded (Table 3). Participants rated the vast majority of the text messages as “helpful”, “likeable”, and “motivating to quit”. Text messages that contained tips about how best to use pharmacologic therapy were rated the lowest, whereas text messages about the health benefits of quitting were rated the highest.

**Table 3:** Participants’ ratings and reactions to text messages

Text message	Text helpful?		Text likeable?		Text motivating to quit?		Participant Reactions to texts (Representative quote)
	Yes	%	Yes	%	Yes	%	
**Texts for individuals who want to quit**							
1. People, places, and things can make you want to smoke. Write down your triggers. Make a plan to help you steer clear of them on you quit day.	17	85%	17	85%	15	75%	*“If you see a group of people smoking. Oh, let me get a cigarette. But if you have someone texting you like, you got this, you can do it, you’re strong, that’s very helpful.” (P9)*
2. Reminding yourself of your reasons for quitting can help keep you on track when you need a boost. Take a few minutes to revisit your list.	15	75%	15	75%	14	70%	*“I think having and hearing just overall little tips over the period of a day, and little pick-me-ups would help motivate me.” (P16)*
3. Counseling and medication can increase your chances of successfully quitting. Talk to your doctor about the best options for you.	16	80%	13	65%	14	70%	*“Well, even ask them what type of resources they may need, and that would be an even better way to [help].” (P1)*
**Texts to provide encouragement to stay quit**							
4. Stay positive. Do not let things get you down. Your journey to a smokefree life might be a struggle, but looking back it will be well worth it.	18	90%	18	90%	18	90%	*“Because it reminds me, and that way helps me more, to stop. That I know somebody out there cares. It helps me stop.” (P12)*
5. Life knocks you down sometimes but YOU make the choice to get back up. Quitting smoking is no different. Do not look back now.	15	75%	14	70%	15	75%	*“I like these encouragement ones.” (P14)*
6. If you slip, keep trying to quit. Learn from your mistakes this time.	14	70%	14	70%	14	70%	*“I think it’s just so basic, it’s obvious. You know what I mean? It’s just telling you something you already know.” (P17)*
**Texts to motivate individuals to quit**							
7. Quitting can be hard. Think about ways you might be better off if you quit.	17	85%	17	85%	17	85%	*“It took me a while to learn how life can get you, man, how quick it can go. Especially now. It’s a good message.” (P7)*
8. Think of the challenges you’ve overcome in the past. Harness those strengths that got you through to help you quit smoking.	14	70%	15	75%	15	75%	*“Yeah. Motivational messages. Nothing but positive messages is what people need.” (P1)*
9. Deciding to quit smoking is a difficult decision. Every difficult decision has pros and cons. Think about your pros/cons for quitting and where that leaves you.	13	65%	13	65%	13	65%	*“I mean, just to get a message like that, and to know someone cares enough to send that, and for you to read it, and really think about what you’re doing’.” (P19)*
10. Thought for the day: how would your life get easier if you didn’t smoke?	19	95%	18	90%	19	95%	*“Some of them would be very motivating, Keep me motivated through the day.” (P10)*
11. Thought for the day: what would you be able to do if you didn’t smoke?	18	90%	18	90%	18	90%	*“I just think the positive affirmation stuff [is helpful]. If you say, “You can go on a great vacation.” Show them how much a vacation costs. Then show them what they could be getting, I guess.” (P17)*
12. Quitting smoking is stressful but continuing to smoke is also stressful—think of the cost, the addiction, health problems, etc. How does smoking stress you out?	15	75%	15	75%	15	75%	*“They would motivate me to stop smoking every day.” (P4)*
13. Thought for the day: how would your life be different 1 year from now if you quit smoking? What about 5 years?	17	85%	17	85%	16	80%	*“I’d feel good about quitting ‘cause, man, at least I know someone’s thinking about me, man, to send me a text.” (P11)*
**Texts on health risks of smoking**							
14. In the U.S., tobacco kills more people than AIDS, alcohol, car accidents, murders, suicides, drugs, and fires combined. Quit today!	17	85%	16	80%	17	85%	*“The stuff they put, like death and destruction it causes. I don’t need to hear about it anymore. I’ve always believed in more positive reinforcement of stuff than negative.” (P16)*
15. Do not be fooled! There is no such thing as a safe cigarette. Quitting is the only way to protect yourself from the health risks of smoking.	13	65%	12	60%	13	65%	*“That’s actually kind of annoying. You’re tellin’ me things I’ve already known.” (P2)*
**Tips to address mistrust and misconceptions of medications to treat tobacco dependence**							
16. Nicotine patch, Wellbutrin, or Chantix double your chances of quitting smoking and do not increase the risk of heart attacks	14	70%	14	70%	14	70%	*“Yeah, I would like that. It’s be a good reminder.” (P4)*
18. The state of Massachusetts offers medications to help you quit smoking at a low or no cost. Call the MA quitline at 1-800-QUIT-NOW for more information.	13	65%	12	60%	13	65%	*“I don’t like the messages like they’re just normal. The 1-800—It’s helpful, but it’s always the same thing. That’s always around, that message.” (P14)*
**Tips to Increase Clinical Utility of Medications**							
19. Take dose of Chantix after eating and with a full glass of water to limit nausea	9	45%	8	40%	8	40%	*“I think you should know if the person’s going to be on medications or not. If you knew someone was leaning towards Chantix, that would be their message.” (P1)*
20. Avoid taking Wellbutrin at bedtime to minimize insomnia	11	55%	9	45%	9	45%	*“I disliked the whole “don’t take the medicine before goin’ to sleep …I didn’t like that one because, most of the time, I like to smoke cigarettes before I go to sleep, which is at night. It’s contradicting.” (P2).*
**Texts on the Benefits of Quitting (Substance Use-specific)**							
21. People who quit smoking have higher success in quitting other substances, such as cocaine, heroin, and alcohol.	16	80%	16	80%	16	80%	*“Cause they go hand in hand. I go to meetings and a lot of people that quit substance abuse, they also quit cigarettes.” (P8)*
**Health Benefits of Quitting (General)**							
22. Quitting smoking lowers your blood pressure and heart rate almost immediately.	17	85%	16	80%	16	80%	*“I like that one a lot. I did not know that. That motivates me a lot, cause all it takes is just one [day] for my health to start improving. That’s crazy.” (P14)*
23. After quitting smoking, your risk of a heart attack declines within 24 h of quitting.	17	85%	16	80%	16	80%	*“Everyone knows if you smoke, you should probably quit, but seeing’ something like within 24 h it can reduce many things like that, it’s eye-opening.” (P5)*
24. People who quit smoking after having a heart attack reduce their chances of having another heart attack by half	18	90%	17	85%	17	85%	*“Really? Wow. I didn’t know that.” (P6)*
25. Quitting smoking is the only proven beneficial treatment for reducing progression in mild to moderate/severe COPD.	18	90%	17	85%	17	85%	*“Damn. Ok. I lost my grandmother and my grandfather to smoking. …cause my mother died of smoking.” (P16)*
**Cost Savings of Quitting**							
26. Smoking 1 pack of cigarettes a day for 1 year costs over $3500! Think about all the other things you could be spending that money on.	17	85%	17	85%	17	85%	*“It’s telling me, man, if I didn’t smoke a pack, how much money I can spend on something else.” (P11)*

#### V. Quitting cigarettes while quitting other substances

The last 9 participants enrolled received the additional BQS-SAT questionnaire. Seven (78%; 7/9) reported that it was hard to quit cigarettes while quitting other substances. Seven (78%; 7/9) reported quitting smoking during substance use treatment would make it harder to stay sober from other substances. Seven (78%; 7/9) reported that they smoked to cope with urges to drink or use drugs.

### Qualitative interviews

#### I. Feasibility of text message programs

Most participants did not perceive that cell phone access and service would be a barrier to participating in a text message program:

*“Yeah, I don’t know of any cell phone carriers that don’t do unlimited text messaging for free anyway at this point. I would say I don’t really see any barriers except for personal ones.” (P16)*

However, a few participants reported that they did not always have cell phone access; these participants noted that they would retain the same phone number when they did regain cell phone access:

*“Right now, I’m not working or doing anything. I’m in the hospital, so I can’t pay the bill if I’m in the hospital. ” (P2)*

*“Yeah, I've lost plenty of phones. I usually get [another], but with the same number.” (P13)*

#### II. Acceptability of text message programs

Qualitative analysis demonstrated that participants would be willing to enroll in a text message program for smoking cessation. Many noted that the lack of support in their personal lives was a barrier to tobacco cessation and they were interested in receiving text messages because it would feel like someone cared and that would motivate cessation:

*“It would remind me throughout the day that I’m trying to quit. It’d be like a friend. Some people don’t have friends, so if you don’t connect with anybody, that’s a perfect way.” (P1)*

*“Yeah, it’d be helpful for me to get it ‘cause at least I know that they still caring for me, you understand? The text messages is helping me think, ‘No, don’t go down that road again.’ You know what I’m saying?” (P7)*

Individuals believed that receiving text messages would be useful reminders that could assist in smoking cessation efforts: *“I would feel okay with that [getting text messages]. Just things I didn’t know, and little messages to get throughout the day that kind of stop and make me think about just smoking at all.” (P19).*

Participants who were only a little/not at all interested in receiving text messages about smoking cessation perceived that text messages might be difficult to understand: *“You [are] texting the wrong man with something like that, you know what I mean? For one, I don’t care about it, but two I don’t understand it.” (P7).*

Some individuals who had only a little interest in receiving text messages because they were not yet ready to quit believed that such a program could help motivate them in the future: *“[Text message programs] might be good. Annoying at first because I’m not ready to quit, but when the time comes…” (P5).*

Others believed that for individuals not motivated to stop smoking, receiving messages about smoking cessation would be neither helpful nor useful: *“Well, they [people] might not want to hear [such messages], if they smoking cigarettes, and then they’d probably cut it off in the message, if they’re smoking a cigarette” (P4).*

#### III. Program content: Response testing, feedback to text messages and content suggestions

Based on our pre-specified social-contextual model, we probed in semi-structured interviews for barriers and facilitators to smoking cessation and how text messages may or may not be helpful in addressing these factors (Table 3).

##### Messages about avoiding triggers

Participant’s environment was commonly stated as a reason why many participants smoked: *“The only time I smoke cigarettes [is] when I see people around me that’s smoking, it triggers. It just makes me want to smoke.” (P12).*

Messages about managing these triggers were particularly helpful: *“It (text message) would motivate me, man, by reminding me that you need to be careful. Be careful, ‘cause you might get around a group of people who smoking and you don’t want to smoke, so you try to keep your distance.” (P11).*

##### Messages about concurrent opioid and cigarette quit efforts

Some patients discussed how it would be difficult to quit opioids and cigarettes together: *“I know in the past, I’ve always used as an excuse to say, ‘I don’t want to quit too much things at once and put too much pressure on myself,’ but I think that was more of an excuse than anything.” (P10).*

For many participants, stopping heroin was their priority and hearing the message that quitting cigarettes could help them remain abstinent from opioids was particularly motivating: *“I liked the one that meant things, ‘a lot of people that stop smoking cigarettes, it helps them to stop doing a lot of other drugs.’ Yeah, I’m going to try.” (P2).*

##### Messages about the cost-savings associated with stopping smoking

Participants talked about the high monetary cost of smoking cigarettes: *“First and foremost, the price of cigarettes is expensive and that’s kind of hard to deal with” (P8).*

Most participants found messages on cost-savings helpful: *“The cost saving one- that’s a good one. I think that’s helpful. You can go on a great vacation. Show ‘em how much a vacation costs. Then show ‘em what they could be getting (by stopping).” (P17).*

##### Messages about the health benefits of quitting smoking

Most participants understood tobacco-related health effects: *“The cigarette is a drug too-for me, it’s a sickness and a drug just like heroin and cocaine. They all have effect of your health, and they all can kill you or make your life shorter. If they can do that for people that are using drugs like, hey, listen, I feel like I need somebody to talk to—it should be the same thing cigarette-wise.”*

But there were some participants who did not realize the serious health effects of tobacco use: *“I just don’t understand it. I thought a heart attack come from too much working or too much stressing out, not just sitting back, taking a drag off a cigarette, you understand? (P7).*

The vast majority learned from the content on health benefits and perceived these messages to be helpful: *“I mean, everyone knows if you smoke, you should probably quit, but seeing something like within 24 hours it can reduce many things like that, it’s eye-opening.” (P5).*

##### Messages about the health risks of smoking

On the other hand, many noted that they knew the negative health effects of smoking cigarettes and hearing these facts did not motivate them to stop smoking, indicating that they preferred messages about the health benefits of tobacco cessation: *“Every adult, unless there’s something wrong with you or you’ve been living somewhere in the woods for the last 30 years knows the dangers of smoking. I already know it. I don’t need to hear about it anymore. What I would rather hear is stuff like, ‘After quitting smoking, your risk of heart attack declines within 24 hours after quitting.’ I think having and hearing the health benefits and the cost savings and just overall little tips over the period of a day, and little pick-me-ups would motivate me” (P16).*

##### Messages about addressing mistrust in medications

Some participants discussed the benefits of taking varenicline (Chantix) to stop smoking: *“The Chantix- it just made you not want a cigarette. I mean, it didn’t even cross your mind to want a cigarette. I’d smoke weed even less, too. I didn’t want nothing to drink. It was pretty good. I like the Chantix.” (P1).*

One participant had mistrust with varenicline and the messages did not assuage that mistrust: *“It (text message) is saying these medications do not increase the risk of suicide or psychiatric events. That’s a lie, so. There’s tons of studies on Chantix and Wellbutrin, ‘cause I went through it myself. It caused psychiatric problems with me. That’s lying.” (P17).*

#### IV. Recommended features to include in building future text message program

Further recommendations from interviews regarding features of the smoking cessation text message program included (1) preference for positive and encouraging tone of messages, (2) interactivity, (3) delivering personalized messages as opposed to generic messages, (4) tailoring message frequency and program duration to individual preferences, (5) provision of links to local resources where individuals can find information about substance use treatment, tobacco treatment, and unmet SDOH needs, and (6) individualizing timing of intervention delivery.

##### Participants prefer the tone of messages to be positive and encouraging

The importance of positively framed messages was noted across interviews, with participants frequently mentioning that encouraging messages such as *“you got this, you can do it, you’re strong,” (P9)* would help them in their smoking cessation efforts:

*“I think more like the empowerment is good…I think a lot of people are knowledgeable. They just need more of the empowerment and stuff to quit.” (P17)*

*“Nothing but positive messages is what people need” (P1)*

##### Participants would like there to be interactive features

Participants suggested that having interactive features (e.g. responding to cravings) when and if needed, would enhance the program: *“If I was able to text like, ‘hey, I’ve had a stressful day. I feel like the nicotine patch is not working’. Then, there’s always somebody 24 hours or something’. ‘Hey, just hang in there.’ You know what I mean?” (P2).*

##### Participants expressed not wanting generic messages

Some individuals noted that they would not like generic messages: *“Other ones (messages) didn’t really apply and I think would make me not want to even look at the messages sometimes, because I would think they were just generic messages and it didn’t really care if I quit it or not, just we’re throwing stuff out there. (P10).*

##### Participants preferred a tailored approach regarding frequency and duration of text messages

Most participants discussed that they would like to receive daily text messages, ranging from 1 to 3 texts per day for at least a month, though many stated they would like to continue receiving messages *“until I quit” (P14)*. Some noted that they preferred if the program frequency and duration were tailored to each specific participant:

*“I know once a morning,—it depends on where you are. The person should have the option of a matter of once a day, a matter of twice a day, or matter of once a week. Because some people get frustrated if they’re not really there, you know what I’m saying?” (P1)*

*“I’d say maybe when you set it up you can ask the person how they want it. I think every person’s different. Maybe they can do it like how they want.” (P17)*

##### Participants would like assistance with managing stress and provision of resources for unmet SDOH needs and OUD

Many participants discussed how they wanted messages about *“how to manage stress, everyone has stress” (P10).* Several participants additionally discussed that they wanted resources about unmet SDOH needs (housing, food insecurity, employment opportunities) and where to get help for their OUD, since these factors contributed to their reasons for smoking: *“Yeah. You can [include those messages] it’s hitting a certain demographic, obviously. A lot of times those people [patients with OUD]—it’s commonality needs.” (P17).*

When asked about how they would like to receive information about resources for SDOH and OUD treatment, two participants responded that providing links would be helpful: *“Ask them what type of resources they may need, and put a link. That would make it easier. Just throw a couple of links in it, and we can just press it. Because the hardest thing for me is to find (resources).”(P1).*

##### Participants discussed that the timing of smoking cessation interventions needs to be individualized

Many participants described the best time for focusing on stopping cigarettes was when they were in treatment for OUD, mostly because they had less desire to smoke cigarettes when they were not using opioids:

*“I really don't like cigarettes without getting high, so I think that if I didn't get high, I could quit easier. That's how I feel about that.” (P8)*

*“I have no idea what they have in suboxone that makes you not get the same high, but whatever they have in it doesn’t make you want to smoke cigarettes like it does when you get high off of heroin or other stuff.” (P14)*

Others, discussed how they preferred incremental quitting: *“Cause I would never be able to just quit all at once due to nerves. Some people have anxieties, some people are PTSDs, some people have this, that. It’s too much all at once I feel for me.” (P15).*

## Discussion

The current study demonstrates that individuals with OUD who smoke cigarettes are interested in receiving text messages for smoking cessation or to help motivate them to quit. Our findings demonstrate that a text message program to promote smoking cessation in individuals who have OUD is feasible in that individuals participating in this study owned cell phones, had unlimited text message plans, and frequently sent and received text messages. There is evidence that computer-tailored programs, which take into account a participant’s smoking behavior and motivations, are among the most effective digital-health strategies to promote smoking cessation [29–31]. Our study demonstrates that individuals with OUD are receptive to such personalized digital health interventions and support the findings of *Langdon* et al. that individuals with OUD prefer motivational and interactive text-messaging programs that are aimed to improve treatment outcomes [14]. Participants discussed that text messages would be helpful not only because they are convenient and accessible, but because receiving text messages would overcome the lack of social support experienced by many of these participants. Individuals provided valuable feedback on message content, tone, timing and framework as discussed below.

We demonstrate that almost all participants prefer gain-framed (benefits of quitting smoking) rather than loss-framed (harms of continuing to smoke cigarettes) messages. Participants discussed how they already knew the harms of smoking, and enjoyed learning new information on the health benefits of quitting. Participants stressed the importance of positively framed messages and discussed how the messages should provide encouragement. Historically, most antismoking messages have emphasized the costs of not quitting smoking, including the CDC’s *Tips From Former Smokers® (Tips®)* anti-smoking ad campaign that highlights the real-life consequences of smoking. Yet several studies suggest smoking cessation messages that highlight gains associated with stopping smoking rather than losses associated with continuing to smoke are more effective at promoting smoking cessation [32–34]. Our findings add to the growing body of evidence that individuals with tobacco dependence, including those with concurrent OUD, have positive perceptions of gain-framed messages.

There is growing evidence that co-treatment of OUD and tobacco use disorder can lead to reduced drug use and sustained recovery and does not have a negative impact on substance use outcomes [35]. Still, many treatment programs do not focus on tobacco use given the belief that this is of lower priority and could jeopardize other quit efforts [2]. Our findings support the belief that individuals with OUD are open to, and may even prefer, receiving co-treatment. Nevertheless, it is notable that participants felt it was important to prioritize individual preference as to when the intervention is delivered. Thus, a future text message program could be a viable intervention to offer during substance use treatment programs as a co-treatment option or a post-OUD treatment option.

Participants additionally discussed how text messages that addressed common barriers that individuals with OUD face, such as triggers in their environment, stress, unmet SDOH needs, and use of other substances would be helpful in promoting cessation. Suggestions included providing links to community resources for unmet SDOH needs (e.g. food pantries) and OUD. Given the high percentage of Medicaid-insured, unemployed, and homelessness among participants, it was not surprising that participants discussed how resources to address these unmet SDOH needs that could serve as barriers to quitting smoking, were found to be helpful. To capitalize on participants’ preference for personalized messages, future text message programs could consider the addition of an SDOH screener through text message [36]; identified unmet social needs could then be responded to with links for resources.

There are strengths and limitations of the study. The strengths include a focus on an understudied medically vulnerable population with high smoking rates and numerous barriers to stopping smoking, including unmet social needs, and use of quantitative and qualitative methods to help design a tailored and engaging intervention to address these factors. The study limitations included a small sample size and recruitment from one safety-net hospital, which limits generalizability.

Findings from this study provide valuable insight into content and features to include in development of text message programs to address barriers to smoking cessation in individuals who have OUD and smoke cigarettes. Key take-away points for program development included: 1) content should focus on the health-benefits of quitting (both general and opioid-specific) rather than the health-risks of continued smoking, 2) the tone of messages should be positive and up-lifting, and 3) the frequency of text messages should be at least once per day and the duration for at least 1 month. Given that individuals with OUD who smoke cigarettes are less likely to receive tobacco treatment, content on the benefits of pharmacotherapy in promoting smoking cessation and messages that address mistrust and misconceptions of pharmacotherapy is important to consider, despite mixed perceptions on these type of messages. Future work will seek to evaluate the feasibility and acceptability of a text message program informed by these results.

## Conclusions

Individuals with OUD who smoke cigarettes perceive that a text message program designed to promote smoking cessation would motivate and support smoking cessation efforts. Such a program would be feasible in this population, and text messages should include content on addressing SDOH barriers that this population faces.

## Data Availability

The data collected and analyzed during the current study are available from the corresponding author on reasonable request.

## Abbreviations

OUD: Opioid use disorder
OAT: Opioid agonist therapy
SDOH: Social determinants of health
BQS-SAT: Barriers to Quitting Smoking in Substance Abuse Treatment
